# Social isolation in COVID-19: a comparative study between Korea and Vietnam

**DOI:** 10.1186/s12889-023-16491-0

**Published:** 2023-08-16

**Authors:** Hyeon Jo, Eun-Mi Baek

**Affiliations:** 1 HJ Institute of Technology and Management, 71 Jungdong-Ro 39 104-1602, Gyeonggi-Do 14721 Bucheon-Si, Republic of Korea; 2 Department of Preventive Medicine, College of Medicine, Catholic University of Korea, 222 Banpo-daero, Seocho-Gu, 06591 Seoul, Republic of Korea

**Keywords:** Social isolation, COVID-19, Social distancing, Risk perception, Cross-cultural analysis

## Abstract

Amidst the ongoing COVID-19 pandemic, social isolation has become a pressing issue worldwide, deeply affecting individuals’ mental and physical well-being. This study introduces a theoretical model to understand the factors influencing social isolation in the context of this global health crisis. We employed a survey methodology, collecting data from Korean and Vietnamese university students through a Google survey form. The theoretical model was evaluated using structural equation modeling (SEM), and multi-group analysis (MGA) was used to assess differences between the Korean and Vietnamese student groups. The investigation centered on affective risk perception, cognitive risk perception, social distancing attitude, social distancing intention, and demographic factors like age and gender. Our findings revealed that affective and cognitive risk perceptions have significant positive impacts on attitudes toward social distancing. Furthermore, attitudes towards social distancing were found to significantly influence social distancing intentions. Interestingly, social distancing intention was found to have a significant positive correlation with social isolation. Lastly, demographic factors such as gender and age were found to be significant factors influencing social isolation. Specifically, gender had a positive association, while age showed a negative correlation with social isolation. Moreover, our MGA results showed that the relationship between social distancing intention and social isolation significantly differed between the Korean and Vietnamese student groups, indicating potential cultural or societal influences on this relationship. Such understanding could inform policies and strategies aimed at mitigating the adverse effects of social isolation in the wake of global health crises.

## Introduction

The emergence and spread of the novel coronavirus (COVID-19) across the globe have had extensive implications, beyond physical health, on people’s psychological well-being [5]. Among the numerous psychological consequences of the pandemic, the rise of social isolation—a product of measures such as lockdowns, quarantines, and social distancing—has emerged as a significant issue, affecting large swathes of the global population [48]. Social isolation, characterized by minimal contact with others and a lack of sense of belonging, can lead to serious mental health problems if not properly addressed [45]. Understanding the factors that influence social isolation during the pandemic is thus paramount. This study focuses on university students in South Korea and Vietnam, two countries that have implemented different strategies to manage the pandemic. The young adult population, particularly university students, has been significantly affected by the pandemic. Closures of universities and transitions to online learning have exacerbated feelings of isolation [8, 40]. University students represent a significant portion of the young adult population. While they are not typically seen as a high-risk group for COVID-19, they face unique challenges. The shift to online learning, the inability to socialize, and the uncertainty surrounding their academic and professional futures could have severe implications for their mental well-being [18, 62, 63]. These factors make them a crucial demographic for this research.

Our study proposes a theoretical model to identify the factors influencing social isolation, considering both psychological and demographic factors. Psychological factors such as affective risk perception, cognitive risk perception, social distancing attitude, and social distancing intention are examined, following theories of risk perception and behavior [3, 55]. These factors are relevant given the context of a pandemic, wherein individuals’ perceptions of risk and their attitudes and intentions toward social distancing measures could play significant roles in their experience of social isolation [6, 40, 60, 64, 66, 67]. Demographic factors, specifically gender, and age, are also considered, as existing literature indicates variations in experiences of isolation across different demographic groups [38, 45]. Gender differences in emotional experiences and responses are well documented [17, 25], while age might determine the resources available to cope with isolation and the capacity to transition to digital modes of socialization [54]. Thus, considering these variables could provide a more nuanced understanding of social isolation. Additionally, we conduct a multi-group analysis (MGA) between South Korea and Vietnam. This comparative approach can offer valuable insights into cultural differences, as societal norms around social interactions could influence experiences and perceptions of social isolation.

Despite the substantial body of research on social isolation, there is a noticeable gap in understanding it in the unique context of a pandemic. Moreover, the cross-cultural analysis of this phenomenon remains limited. Our study aims to address these gaps, offering a novel contribution to the discourse surrounding social isolation during a pandemic. Specifically, this research aims to investigate the effects of risk perception, social distancing attitudes and intentions, and demographic factors on social isolation among university students in South Korea and Vietnam.

This paper is structured as follows: The next section provides a detailed review of the relevant literature and the development of hypotheses. It is followed by a discussion of the research methodology employed. The subsequent section presents the results and findings of the study. This is followed by a discussion of the results. Finally, the paper concludes with a discussion of the theoretical contributions, practical implications, limitations, and directions for future research.

## Related work and hypotheses development

The impact of the COVID-19 pandemic on social isolation is a complex phenomenon. It is influenced by various factors including risk perceptions, social distancing attitudes and intentions, and demographic variables.

Risk perception, generally defined as individuals’ subjective judgment about the likelihood of negative events, plays a critical role in understanding the behavior of individuals during a pandemic [55]. It is often categorized into affective and cognitive components. Affective risk perception refers to individuals’ emotional responses to a perceived threat, while cognitive risk perception relates to rational assessments of that threat [41]. Pandemics like COVID-19 pose both physical and psychological risks, which are perceived and responded to differently by individuals [16, 24, 26]. An individual’s perceived risk, both affective and cognitive, can greatly influence their behaviors, including adherence to recommended preventative measures such as social distancing [27]. Moreover, those who perceive a high level of risk may be more likely to isolate themselves, even to the extent of experiencing social isolation [19, 32].

The theory of planned behavior (TPB), developed by [3], has been widely applied in studies predicting human behavior and has shown its relevance in the context of the COVID-19 pandemic as well. According to this theory, attitudes towards behavior (in this case, social distancing attitude), subjective norms, and perceived behavioral control together shape an individual’s behavioral intentions, ultimately influencing their actual behavior. The role of social distancing attitude in determining social distancing intention during the pandemic, which is implied by the TPB, has been confirmed by several studies [1, 33, 36]. Social distancing attitude refers to an individual’s evaluation of social distancing as a behavior. That is, it captures how favorably or unfavorably a person views the act of practicing social distancing. Research suggests that positive attitudes towards social distancing, which could be fostered by understanding its benefits in curbing the spread of the virus, are associated with higher intentions to adhere to social distancing guidelines [11, 20]. Social distancing intention, on the other hand, refers to the degree of an individual’s willingness to engage in social distancing. It reflects a person’s motivation or determination to carry out the behavior. [3] argues that intention is the most immediate determinant of behavior. This suggests that individuals with stronger intentions to practice social distancing are more likely to carry out the behavior consistently. Empirical studies have indeed found a positive relationship between individuals’ intention to practice social distancing and their actual social distancing behavior [9, 47]. The linkage between social distancing attitude and intention suggests that interventions aiming to promote social distancing behavior during the COVID-19 pandemic could benefit from strategies that positively shape individuals’ attitudes towards social distancing, thereby increasing their intentions to perform this behavior.

Demographic factors such as age and gender are often associated with different coping strategies and mental health outcomes during pandemics [62]. For instance, women have been found to experience higher levels of loneliness during the pandemic [31], while younger individuals were more likely to feel isolated due to the lockdown measures [15].

**Fig. 1:**
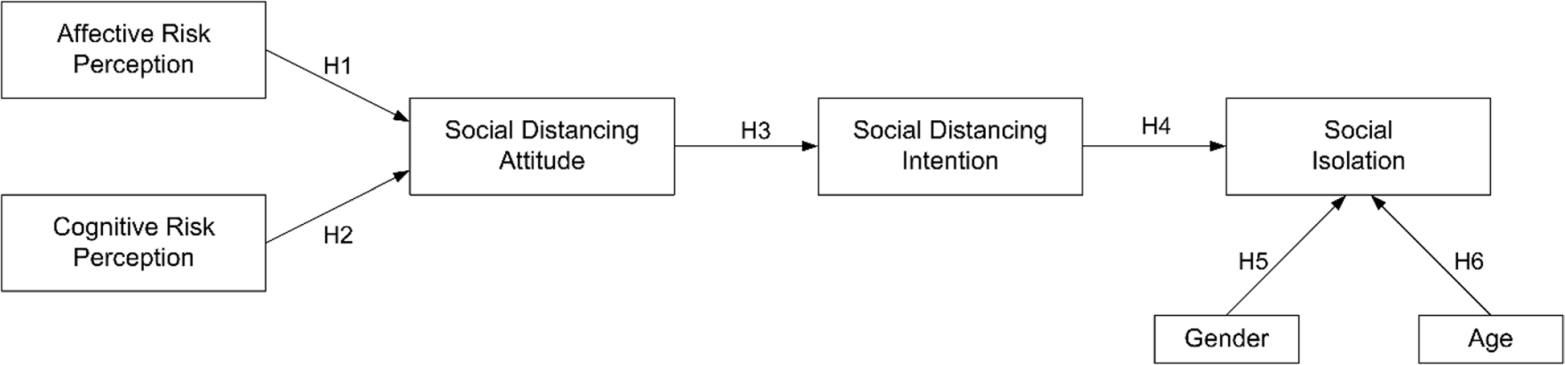
Research framework

Figure 1 shows the research model. This research examines the relationships between affective risk perception, cognitive risk perception, social distancing attitude, social distancing intention, and social isolation, using demographic factors (age and gender) as control variables. The research model is grounded in the Theory of Planned Behavior, which suggests that attitudes and intentions toward a specific behavior (here, social distancing) significantly influence the execution of that behavior. The relationships between these constructs were examined for potential differences between South Korean and Vietnamese university students, enabling an MGA to understand the cultural contexts impact.

### Affective risk perception

Affective risk perception refers to an individual’s emotional response to perceived threats or risks, such as fear, worry, or concern [41]. University students, a population particularly susceptible to the negative consequences of infectious diseases like COVID-19, are more likely to engage in preventative behaviors if they perceive a higher affective risk associated with the disease [13]. Affective risk perceptions have been demonstrated to drive preventive health behaviors in previous research. For instance, Dryhurst et al. [27] found that affective responses to COVID-19 significantly predicted individuals’ self-reported compliance with public health guidelines, including social distancing. Similarly, in a study examining H1N1 influenza preventive behavior, individuals who reported greater worry were more likely to engage in preventative behaviors [52]. Hence, it is reasonable to hypothesize that higher affective risk perception would motivate students to adopt attitudes supportive of social distancing, a key preventive measure against COVID-19. Thus, this study suggests the following hypothesis.

- H1. Affective risk perception positively influences social distancing attitude.

### Cognitive risk perception

Cognitive risk perception pertains to an individuals objective understanding or estimation of the probability of a harmful event occurring [56]. Research has repeatedly demonstrated that cognitive risk perception is a significant factor influencing health-related behaviors. For instance, during the 2009 H1N1 pandemic, individuals who cognitively understood the risks associated with the virus were more likely to engage in preventive behaviors [52]. Similarly, a study by Dryhurst et al. [27] found that cognitive risk perception was associated with compliance with COVID-19 preventative measures, including social distancing. These findings provide compelling evidence that cognitive risk perception may play a role in shaping attitudes toward social distancing. Given the need for continued social distancing to curb the spread of COVID-19 and the pivotal role of cognitive risk perception in informing preventive behaviors, this paper hypothesizes a positive influence. Thus, this study suggests the following hypothesis.

- H2. Cognitive risk perception positively influences social distancing attitude.

### Social distancing attitude

Attitudes, the independent variable, reflect an individual’s favorable or unfavorable evaluations of behavior [3]. In the context of health behavior, the TPB posits that attitudes toward a specific behavior significantly predict the intention to engage in that behavior [3, 43]. Applying this to social distancing, a positive attitude towards social distancing (viewing it as beneficial, responsible, and necessary) is likely to lead to a stronger intention to engage in such behavior. Studies during the COVID-19 pandemic have supported this, indicating that positive attitudes toward social distancing are associated with higher intentions to practice social distancing [1, 22, 30]. Therefore, it is reasonable to hypothesize that social distancing attitudes would influence social distancing intentions. Thus, this study suggests the following hypothesis.

- H3. Social distancing attitude positively influences social distancing intention.

### Social distancing intention

Social distancing intention refers to an individual's planned efforts to maintain physical distance from others to minimize disease transmission risk [3]. Intentions are the most immediate and important determinant of behavior according to the TPB, suggesting that people who intend to perform behaviors are more likely to execute them [3]. Translating this to social distancing, individuals with stronger intentions to practice social distancing would likely follow through with such behavior more consistently, potentially leading to increased social isolation. Aligned with this, studies during the COVID-19 pandemic have shown that adherence to social distancing guidelines can lead to feelings of social isolation [46, 59]. Given these findings, it is plausible to hypothesize that higher social distancing intentions would result in increased social isolation. Thus, this study suggests the following hypothesis.

- H4. Social distancing intention positively influences social isolation.

### Gender and age

Gender and age are critical demographic factors often associated with the experience of social isolation. Gender may play a role in social isolation, with some studies suggesting that women report higher levels of social isolation than men [42]. Age is also an important predictor of social isolation. Older individuals, especially those over the age of 65, tend to experience higher levels of social isolation [58]. This increased risk among older adults may be due to various factors such as the loss of social networks, physical mobility issues, and health problems. These relationships suggest that both age and gender could significantly influence the experience of social isolation, and this has been supported by empirical evidence [21, 23]. Thus, this study suggests the following hypothesis.

- H5. Gender significantly influences social isolation.
- H6. Age significantly influences social isolation.

## Research methodology

### Instrument development

The instrument for this study was developed through several phases, ensuring the validity and reliability of the measurements. First, a literature review was conducted to identify established scales that measured the constructs of interest: affective risk perception, cognitive risk perception, social distancing attitude, social distancing intention, and social isolation. The items from these scales were adapted to fit the context of COVID-19, as seen in Table 7 in Appendix.

For affective and cognitive risk perception, items were adapted from the scales developed by Brug et al. [14]. For social distancing attitude and intention, items were adapted from studies by Afe and Ogunsemi [2], Azodo and Ogbebor [7], and Williams et al. [65]. Lastly, items measuring social isolation were adopted from the study by Raza et al. [51].

The adapted items were then tested in a pilot study with a smaller sample of students. Based on the pilot study, revisions were made to improve the clarity and relevance of the items. The revised survey was then translated into Korean and Vietnamese, and back-translated to ensure linguistic accuracy. Before data collection, the survey was reviewed by several professors for content validity, ambiguous expressions, and logical arrangement. All items were measured on a 7-point Likert scale, ranging from 1 (strongly disagree) to 7 (strongly agree). Demographic information including nationality, gender, and age was also collected. All procedures involving human participants were following the ethical standards of the institutional research committee.

### Sampling and data

The online survey was distributed through various academic and social platforms to reach the target demographic of university students in both Korea and Vietnam. University students represent a demographic that is critical to understanding the impact of COVID-19, and they are an essential group to consider when developing strategies for promoting social distancing and mitigating social isolation. As a population that typically lives, studies, and socializes in close-knit communities, university students are particularly affected by social distancing measures and are at increased risk of social isolation. Furthermore, focusing on university students in both Korea and Vietnam allows for a comparative perspective. Data for this study were gathered through an online survey utilizing Google Forms, an accessible tool enabling straightforward data compilation and subsequent analysis. The data collection period spanned from June 11, 2021, to September 27, 2021. At this juncture, South Korea had implemented nationwide social distancing measures at levels 3 or 4, the most stringent tiers, while Vietnam was under a comprehensive social lockdown. In South Korea, operations of all multi-purpose facilities such as private academies, reading rooms, and gyms were restricted until 10 PM [10]. Schools had transitioned entirely to remote learning. In contrast, Vietnam allowed only essential outings such as purchasing food and medicines [57]. Its schools were also conducting all classes remotely. This particular context greatly impacted the daily routines and social activities of university students, potentially intensifying their experiences of social isolation. Studying the perceptions and behaviors of university students in these two contexts can provide valuable insights into how different public health strategies influence individual attitudes and behaviors related to social distancing and social isolation. The survey questionnaire was designed to understand students' perceptions, attitudes, and intentions towards social distancing and their experiences of social isolation during this period.

The sampling method employed for this study was convenience sampling, a non-probability sampling method that targets an easily accessible and available group of people. This method was appropriate considering the restrictions and safety measures brought about by the COVID-19 pandemic, which has limited face-to-face interactions and enhanced the relevance of online data collection. Several university professors aided in data collection, allowing for a broader reach within the university student population. Professors shared the survey with their students and encouraged participation to ensure a substantial sample size for the study. Although the convenience sampling method may raise questions about the representativeness of the sample, the collaborative efforts of multiple professors and the substantial sample size helped enhance the credibility and generalizability of the findings. This data collection approach, while relatively easy to administer, ensured that a diverse and substantial sample of university students was included in the study. Informed consent was obtained from all participants.

Table 1 shows the details of the sample. In terms of nationality, 180 respondents (37.5%) were Korean, while the majority, 300 respondents (62.5%), were Vietnamese. This multinational representation provides a diverse viewpoint on social distancing and social isolation, which may be influenced by cultural contexts. Regarding gender distribution, there was a considerable difference with 150 respondents (31.3%) identifying as male, and 330 respondents (68.8%) identifying as female. This discrepancy in gender distribution could be an important factor considering the potential gender-based difference in perception and behavior towards COVID-19 and the resultant social distancing and isolation experiences. The sample also encompassed a range of age groups, though the majority of the respondents were relatively young. Specifically, 74 respondents (15.4%) were 19 years or younger, 368 respondents (76.7%) were between the ages of 20 and 23, and 38 respondents (7.9%) were 24 years or older. Given the dynamic nature of the COVID-19 pandemic and its variable impact on different age groups, these demographics provide useful insights into how social isolation and distancing are perceived by younger populations.

**Table 1:** Demographic characteristics of the samples

Demographics	Item	Subjects (*N* = 480)	
		Frequency	Percentage
Nationality	Korea	180	37.5%
	Vietnam	300	62.5%
Gender	Male	150	31.3%
	Female	330	68.8%
Age	19 or younger	74	15.4%
	20–23	368	76.7%
	24 or older	38	7.9%

## Analysis and results

In this study, the partial least squares (PLS) method was employed to address the presence of formative factors and a large number of constructs. PLS is particularly suitable for research involving complex predictive models. It is well-equipped to handle intricate research models that encompass numerous constructs, including formative constructs [35]. To evaluate the reliability, convergent validity, and discriminant validity of the measurement model and structural model, a two-step approach proposed by Anderson & Gerbing [4] was employed.

### Common method bias (CMB)

To assess the potential presence of common method bias in this study, we used Harman's single-factor test [49]. In this test, all variables in the study are loaded onto a single factor in an exploratory factor analysis. If a single factor emerges or one factor accounts for a majority of the covariance among the variables, it indicates a potential CMB issue. The results showed that the single factor accounted for 39.085% of the variance, suggesting that CMB is unlikely to be a substantial concern in our study.

### Measurement model

In examining the measurement model, we assessed reliability, convergent validity, and discriminant validity using various metrics. The reliability of the constructs was examined using Cronbach's alpha and Composite Reliability (CR). As indicated in Table 2, Cronbach's alpha values for all constructs ranged from 0.805 to 0.934, exceeding the recommended threshold of 0.7 [44]. Similarly, the CR values ranged from 0.878 to 0.958, surpassing the acceptable limit of 0.7 [34]. These results confirmed the internal consistency of the constructs.

**Table 2:** Reliability and convergent validity

Construct	Items	Mean	St. Dev.	Factor Loading	Cronbach's Alpha	CR	AVE
Affective Risk Perception	ARP1	5.144	1.766	0.861	0.898	0.928	0.764
	ARP2	5.554	1.568	0.855			
	ARP3	5.481	1.574	0.896			
	ARP4	5.769	1.390	0.883			
Cognitive Risk Perception	CRP1	5.333	1.795	0.925	0.809	0.913	0.839
	CRP2	4.885	1.910	0.906			
Social Distancing Attitude	SDA1	5.619	1.457	0.867	0.805	0.885	0.719
	SDA2	5.531	1.452	0.846			
	SDA3	5.233	1.585	0.830			
Social Distancing Intention	SDI1	6.033	1.193	0.944	0.934	0.958	0.884
	SDI2	5.975	1.218	0.947			
	SDI3	6.004	1.162	0.929			
Social Isolation	SIS1	4.390	1.940	0.796	0.819	0.878	0.645
	SIS2	4.458	1.875	0.711			
	SIS3	4.960	1.877	0.850			
	SIS4	4.650	1.975	0.846			

Convergent validity was evaluated through the average variance extracted (AVE) and factor loadings. All constructs had AVE values above the recommended cut-off of 0.5 [29], indicating substantial convergent validity. Additionally, all items had factor loadings above the threshold of 0.7, supporting the validity of the constructs (Hair et al., 2010).

Discriminant validity, or the extent to which the constructs are distinct, was evaluated through the Fornell-Larcker criterion and the Heterotrait-Monotrait ratio (HTMT). The square root of AVE of each construct (diagonal values in Table 3) was higher than its correlations with other constructs, meeting the Fornell-Larcker criterion. Moreover, the HTMT values (Table 4) were all below the commonly accepted threshold of 0.85 [37], which further supports the discriminant validity of the constructs. In conclusion, the measurement model displayed robust reliability, convergent validity, and discriminant validity, reinforcing the quality of the constructs used in this study.

**Table 3:** Fornell-Larcker scale results

Constructs	1	2	3	4	5
1. Affective Risk Perception	0.874				
2. Cognitive Risk Perception	0.584	0.916			
3. Social Distancing Attitude	0.451	0.397	0.848		
4. Social Distancing Intention	0.387	0.237	0.654	0.940	
5. Social Isolation	0.308	0.424	0.329	0.156	0.803

**Table 4:** HTMT matrix

Constructs	1	2	3	4	5
1. Affective Risk Perception					
2. Cognitive Risk Perception	0.669				
3. Social Distancing Attitude	0.517	0.491			
4. Social Distancing Intention	0.417	0.271	0.751		
5. Social Isolation	0.338	0.510	0.380	0.165	

### Hypothesis test

**Fig. 2:**
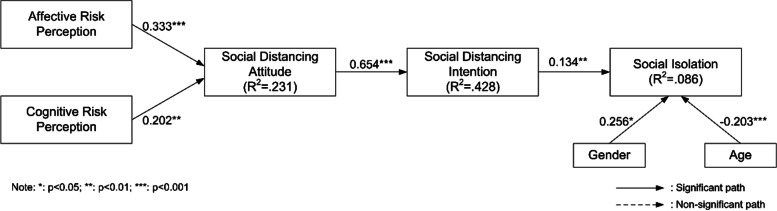
The path coefficients of the research model

The proposed relationships among the constructs were examined using partial least squares structural equation modeling (PLS-SEM). The significance of the path coefficients within the theoretical framework was assessed using the bootstrap resampling method with 5000 resamples. The findings from the analysis are depicted in Fig. 2.

In line with our predictions, affective risk perception shows a significant association with social distancing attitude (b = 0.333, t = 6.903), providing support for H1. Furthermore, crowd perception has a significant positive effect on social distancing attitude (b = 0.202, t = 4.165), supporting H2. As hypothesized, social distancing attitude demonstrates a significant relationship with social distancing intention (b = 0.654, t = 19.455), strongly supporting H3. In line with our predictions, social distancing intention correlates significantly with social isolation (b = 0.134, t = 3.099), supporting H4. Additionally, gender shows a significant influence on social isolation (b = 0.256, t = 2.572), supporting H5. Similarly, age has a significant positive impact on social isolation (b = -0.203, t = 5.513), supporting H6. Table 5 shows the results of hypothesis testing.

**Table 5:** Summary of the results

H	Cause	Effect	Coefficient	T-value	*P*-value	Hypothesis
H1	Affective Risk Perception	Social Distancing Attitude	0.333	6.903	0.000	Supported
H2	Cognitive Risk Perception	Social Distancing Attitude	0.202	4.165	0.000	Supported
H3	Social Distancing Attitude	Social Distancing Intention	0.654	19.455	0.000	Supported
H4	Social Distancing Intention	Social Isolation	0.134	3.099	0.002	Supported
H5	Gender	Social Isolation	0.256	2.572	0.010	Supported
H6	Age	Social Isolation	-0.203	5.513	0.000	Supported

### MGA between Korea and Vietnam

MGA was conducted to examine the differences between the two groups: Korean and Vietnamese students. The purpose of the MGA was to investigate whether the relationships between constructs were significantly different across the two groups.

The results showed a few notable differences between the Korean and Vietnamese samples. Regarding the relationship between social distancing intention and social isolation, there was a significant difference between the two groups, with a two-tailed *p*-value of 0.001. This implies that the impact of social distancing intention on social isolation was significantly higher in the Vietnamese student group compared to the Korean group.

However, the relationships between affective risk perception and social distancing attitude, cognitive risk perception and social distancing attitude, and social distancing attitude and social distancing intention did not show significant differences between the two groups. The same held for the influences of gender and age on social isolation, where no significant differences were found between Korean and Vietnamese students. Table 6 describes the results of MGA.

**Table 6:** MGA (Korea – Vietnam)

H	Cause	Effect	Difference	1-tailed	2-tailed
H1	Affective Risk Perception	Social Distancing Attitude	-0.041	0.655	0.689
H2	Cognitive Risk Perception	Social Distancing Attitude	0.015	0.445	0.890
H3	Social Distancing Attitude	Social Distancing Intention	-0.109	0.956	0.089
H4	Social Distancing Intention	Social Isolation	-0.488	1.000	0.001
H5	Gender	Social Isolation	0.114	0.288	0.575
H6	Age	Social Isolation	-0.099	0.889	0.221

## Discussion

Our findings provide crucial insights into the factors influencing attitudes towards, intentions for, and consequences of social distancing, with a focus on the university student population.

Firstly, our results reveal that affective risk perception significantly influences the attitude toward social distancing. This finding is consistent with previous studies such as those by [14], which suggested that emotional responses to perceived threats are a significant driver of preventive behavior. This means that someone who has a high level of fear or worry about the virus (high ARP) might be more likely to view social distancing positively, considering it as an effective measure to prevent the spread of the virus.

Secondly, cognitive risk perception also demonstrates a significant positive relationship with social distancing attitudes. This aligns with previous research suggesting that a cognitive understanding of risks, including mortality and morbidity rates, can shape attitudes toward preventive behaviors [14, 27, 52]. Hence, a person with a high cognitive risk perception (CRP) might understand the gravity of the situation and therefore may have a positive attitude towards measures like social distancing, recognizing its effectiveness in controlling the spread of the virus. The effects of both affective risk perception and cognitive risk perception on social distancing attitude remained significant across both Korean and Vietnamese student groups, suggesting that these effects are consistent across different cultural contexts. This highlights the importance of both emotional and factual communication in public health campaigns to foster positive attitudes toward preventative measures like social distancing. However, it's important to note that despite these significant relationships, the multi-group analysis (MGA) results showed no significant difference between the Korean and Vietnamese student groups in these relationships. This means that although ARP and CRP significantly influence SDA, the strength of these relationships doesn’t differ significantly between the two national groups.

The third finding suggests a strong link between social distancing attitudes and intentions. This supports the TPB, which posits that favorable attitudes toward a behavior often lead to intentions to perform that behavior [1, 3, 30]. If an individual believes that social distancing is an effective method for controlling the spread of COVID-19 and doesn't perceive the costs (such as inconvenience or feeling of isolation) to outweigh the benefits (like protecting themselves, their family, and the community), they will likely have a positive attitude towards it. Students with a positive attitude towards social distancing are more likely to intend to practice it.

Moreover, our results indicate that social distancing intentions positively influence social isolation. This could be due to the physical and psychological effects of social distancing. As students committed to social distancing measures, they might experience increased feelings of isolation, as reported in studies conducted during the COVID-19 pandemic [12, 46, 59].

This study also revealed a significant difference in how social distancing intentions influence social isolation between the Korean and Vietnamese student populations. Specifically, the Vietnamese student group exhibited a stronger relationship between social distancing intentions and social isolation compared to their Korean counterparts, with a statistically significant two-tailed *p*-value of 0.001. These disparities can be evaluated within the context of the pandemic situation and other socio-political factors prevailing in both countries during the data collection period. Vietnam, for instance, was under a strict social lockdown, allowing only for essential outings like purchasing food or medicines. Conversely, South Korea was enforcing level 3 or 4 social distancing measures, with public facilities operating until 10 PM and schools conducting fully remote classes. It's conceivable that the stricter regulations in Vietnam might have amplified the correlation between social distancing intentions and social isolation among its students. The lockdown measures could potentially intensify the sense of isolation as students strive to adhere to the imposed restrictions. Moreover, cultural factors could have played a role in these differential findings. It's possible that variations in social norms and expectations between the two societies might have affected how students perceive and experience social isolation. Additionally, political aspects, such as the public's trust in government and the effectiveness of its pandemic response, might have influenced students' attitudes towards social distancing and their subsequent experiences of isolation. Hence, this significant difference emphasizes the necessity of taking into account these socio-political and cultural contexts when interpreting the impacts of social distancing intentions on social isolation. It also underscores the importance of designing culturally and contextually sensitive interventions to mitigate the negative effects of social isolation during a pandemic.

Lastly, our findings suggest that gender and age influence social isolation. Females seem more prone to social isolation than males, perhaps due to differential social roles and expectations, aligning with the findings of Victor and Yang [61]. Conversely, age has a negative relationship with social isolation, suggesting that older students might have more effective coping strategies, as reported by Rahman et al. [50]. This might be due to the reduction in their physical interactions with others.

These findings underscore the multifaceted nature of social responses to a pandemic, highlighting the importance of considering both cognitive and affective aspects of risk perception, demographic factors, and the potential mental health effects of preventive measures.

## Conclusion

### Theoretical implications

Our research contributes to the understanding of preventive behavior in the context of a pandemic, particularly in terms of how risk perception influences attitudes and behaviors related to social distancing among university students. While prior research, such as Brug et al. [14], Savadori and Lauriola [53], Jo [40], and Jo [39], has explored the role of risk perception in shaping behaviors, our study delves deeper into the distinctions between affective and cognitive risk perceptions. This nuanced approach enhances our comprehension of how these different aspects of risk perception can independently contribute to attitudes toward social distancing. Previous studies may have overlooked the differential impacts of affective and cognitive aspects of risk perception, which our research highlights. Consequently, scholars should consider both the emotional response and cognitive understanding of risk in their future studies of health-related behaviors.

Additionally, our research extends the TPB [3] by demonstrating its applicability to the context of a pandemic, specifically concerning social distancing. This application contributes to a more comprehensive understanding of how attitudes influence intentions, even in unique and unprecedented circumstances. While Ajzen's original work provided valuable groundwork, our study advances this theory by validating its use in the specific context of a global health crisis. Thus, it provides a clear avenue for scholars to investigate how other health attitudes and intentions might operate within a similar framework during other extraordinary events.

Furthermore, our research uncovers the link between social distancing intentions and social isolation. Existing literature has noted the psychological effects of social distancing [12], but our study is among the first to empirically demonstrate a direct correlation between social distancing intention and social isolation. This link has been under-explored in prior research and may be a critical factor to consider in the study of pandemic-related behaviors and their impacts. Future studies may want to examine how to mitigate the negative effects of social isolation while maintaining strong social distancing intentions.

Our study's exploration of gender and age as influencing factors in social isolation offers a new perspective. While previous studies such as those by Victor and Yang [61] have explored demographic factors concerning loneliness and social isolation, the specific context of a pandemic and social distancing may shape these relationships differently. Our study identifies these critical differences, encouraging scholars to consider how these demographic variables might interact with other factors in shaping social isolation during unprecedented times.

Lastly, the research presents that Vietnamese students, in particular, demonstrated a stronger correlation between social distancing intentions and resulting feelings of social isolation, an observation substantiated. This implies that the intensification of Vietnamese students' commitment to upholding social distancing regulations corresponded to a more heightened experience of social isolation when compared to their Korean peers. This disparity may be attributable to varying cultural, societal, or circumstantial factors across the two nations. Elements such as societal norms, pandemic conditions, as well as the execution and enforcement of social distancing protocols could be contributing factors to this variance. Moreover, how individuals from diverse cultures perceive and respond to social isolation can differ significantly. Societies that traditionally value interconnectedness and frequent social engagements might find that restrictive measures amplify feelings of isolation. Conversely, in societies where individualistic values are more dominant, social distancing could potentially lead to less profound feelings of isolation.

### Implications for practitioners

Firstly, the findings on the positive influence of affective and cognitive risk perceptions on social distancing attitudes suggest that policymakers and health educators should emphasize these two facets in their health communication strategies. They could provide up-to-date and accurate information about the risks of COVID-19 to enhance cognitive risk perception, while also addressing the emotional concerns and fears that people may have about the virus [14]. For instance, conveying personal stories from individuals who have contracted the virus or who have lost loved ones might heighten affective risk perceptions, while explaining the science behind COVID-19 transmission could enhance cognitive understanding.

The results also imply that efforts to promote positive attitudes towards social distancing could potentially enhance the intentions to follow social distancing guidelines. Therefore, health communication strategies should not just focus on providing information about the risk but also encourage a positive view of social distancing measures. For example, campaigns could highlight the societal benefits of social distancing, such as protecting vulnerable community members or reducing strain on healthcare facilities [28, 65]. In this way, social distancing might be seen as a form of civic responsibility, rather than just a personal protective measure.

Our findings that social distancing intentions can lead to social isolation is an important implication for mental health professionals and support services. They should anticipate and prepare for the potential increase in feelings of loneliness and isolation due to extended periods of social distancing [51]. Strategies could include providing online mental health resources, promoting digital social platforms for connection, or even organizing socially-distanced community events. These efforts would aid in mitigating the negative effects of social isolation while maintaining necessary distancing precautions.

The influence of demographic factors like age and gender on social isolation is a critical consideration for policymakers and community organizations. Interventions to reduce social isolation should consider the unique needs and experiences of different age groups and genders. For example, older individuals might need additional technical support to access digital social platforms, while women might require more mental health resources due to their higher reported rates of loneliness during the pandemic [61]. These interventions would provide more targeted support, reducing social isolation effectively in these different demographic groups.

Finally, the multi-group analysis demonstrates a significant difference in the relationship between social distancing intention and social isolation between Korea and Vietnam. This revelation holds significant implications for policymakers, health educators, and mental health professionals. It underscores the necessity to factor in cultural context when formulating health guidelines like social distancing, and when devising plans to mitigate the potential mental health repercussions. Tailoring interventions to cater to the specific needs and lived experiences of various cultural groups may yield greater success in alleviating the adverse effects of social isolation during a pandemic. Therefore, gaining insights into these differences could pave the path for more nuanced, culturally mindful public health interventions in the future. Furthermore, it could stimulate future research to investigate the underlying reasons for these disparities and how they can be effectively addressed.

### Limitation and future research

Despite its contributions, this study has certain limitations which present avenues for future research. Primarily, our research was cross-sectional, capturing respondents' attitudes, perceptions, and behaviors at one specific point in time. Given the fluid nature of the pandemic and subsequent changes in public sentiment, future studies could adopt a longitudinal design to examine how these variables evolve. Secondly, we focused on five constructs related to social distancing and social isolation. There are, however, other relevant factors such as personal beliefs, cultural norms, or the perceived effectiveness of government policies that may influence these outcomes. Further research could incorporate these variables to provide a more comprehensive understanding of the phenomena. Finally, our study used self-reported data, which may be subject to response bias. Future studies could employ alternative data collection methods, such as observational studies or experiments, to cross-validate the findings.

## Data Availability

The data used in this study are available from the corresponding authors upon reasonable request.

## Appendix

**Table 7:** List of constructs and items

Construct	Items	Mean	Reference
Affective Risk Perception	ARP1	I have concerns about the possibility of contracting COVID-19	Brug et al. [14]
	ARP2	I have concerns regarding the potential contraction of COVID-19 by my family members	
	ARP3	I have concerns about the occurrence of COVID-19 in my region	
	ARP4	I have concerns about the emergence of COVID-19 as a health concern	
Cognitive Risk Perception	CRP1	The probability of contracting COVID-19 is significantly higher compared to other diseases	Brug et al. [14]
	CRP2	The likelihood of mortality from COVID-19 is high	
Social Distancing Attitude	SDA1	In my perspective, implementing social distancing measures will have a positive effect on controlling the spread of COVID-19	Afe and Ogunsemi [2], Azodo and Ogbebor [7], and Williams et al. [65]
	SDA2	The utilization of social distancing is advantageous for patient care	
	SDA3	I find the implementation of social distancing for COVID-19 control to be intriguing	
Social Distancing Intention	SDI1	I intend to practice social distancing when it proves effective in preventing COVID-19	Williams et al. [65], Fong et al. [28]
	SDI2	I have the intention to utilize social distancing when it is necessary to achieve favorable outcomes in avoiding COVID-19	
	SDI3	I am committed to practicing social distancing for the well-being of both myself and others	
Social Isolation	SIS1	I felt alone and friendless during social distancing/lockdown	Raza et al. [51]
	SIS2	I felt isolated from other people during social distancing/lockdown	
	SIS3	I do not have someone to share my feelings with during social distancing/lockdown	
	SIS4	I found it difficult to get in touch with others when I needed others to feel they had to help me during social distancing/lockdown	

## References

[1] Adiyoso W, Wilopo W (2020). Social distancing intentions to reduce the spread of COVID-19: The extended theory of planned behavior. BMC Public Health.

[2] Afe TO, Ogunsemi O (2016). Social distancing attitudes toward the mentally ill and victims of sexual violence among college students in Southwest Nigeria. Indian J Soc Psychiatry.

[3] Ajzen I (1991). The theory of planned behavior. Organ Behav Hum Decis Process.

[4] Anderson JC, Gerbing DW (1988). Structural equation modeling in practice: A review and recommended two-step approach. Psychol Bull.

[5] Armour C, McGlinchey E, Butter S, McAloney-Kocaman K, McPherson KE (2021). The COVID-19 psychological wellbeing study: understanding the longitudinal psychosocial impact of the COVID-19 pandemic in the UK; a methodological overview paper. J Psychopathol Behav Assess.

[6] Ayran G, ÇevikÖzdemir HN, Yaman E (2023). The effect of risk perception, mask use, and social distance behavior on perceived stress in the COVID-19 process: A sectional study. J Child Adolesc Psychiatr Nurs.

[7] Azodo CC, Ogbebor OG (2019). Social distance towards halitosis sufferers. Swiss Dent J.

[8] Baltà-Salvador R, Olmedo-Torre N, Peña M, Renta-Davids A-I (2021). Academic and emotional effects of online learning during the COVID-19 pandemic on engineering students. Educ Inf Technol.

[9] Barari S, Caria S, Davola A, Falco P, Fetzer T, Fiorin S, Hensel L, Ivchenko A, Jachimowicz J, King G. Evaluating COVID-19 public health messaging in Italy: Self-reported compliance and growing mental health concerns. medRxiv. 2020. 10.1101/2020.03.27.20042820.

[10] BBCNewsKorea. COVID-19: Phase 4 in the National Capital Region for 12 days and 2 weeks... What’s different? BBCNewsKorea. 2021. Retrieved July 25th from https://www.bbc.com/korean/news-57794898.

[11] Bogg T, Milad E (2020). Demographic, personality, and social cognition correlates of coronavirus guideline adherence in a US sample. Health Psychol.

[12] Brooks SK, Webster RK, Smith LE, Woodland L, Wessely S, Greenberg N, Rubin GJ (2020). The psychological impact of quarantine and how to reduce it: rapid review of the evidence. Lancet.

[13] Brouard S, Vasilopoulos P, Becher M (2020). Sociodemographic and psychological correlates of compliance with the COVID-19 public health measures in France. CJPS/RCSP.

[14] Brug J, Aro AR, Oenema A, De Zwart O, Richardus JH, Bishop GD (2004). SARS risk perception, knowledge, precautions, and information sources, the Netherlands. Emerg Infect Dis.

[15] Bu F, Steptoe A, Fancourt D (2020). Who is lonely in lockdown? Cross-cohort analyses of predictors of loneliness before and during the COVID-19 pandemic. Public Health.

[16] Chan EYY, Huang Z, Lo ESK, Hung KKC, Wong ELY, Wong SYS (2020). Sociodemographic predictors of health risk perception, attitude and behavior practices associated with health-emergency disaster risk management for biological hazards: the case of COVID-19 pandemic in Hong Kong, SAR China. Int J Environ Res Public Health.

[17] Chaplin TM (2015). Gender and Emotion Expression: A Developmental Contextual Perspective. Emot Rev.

[18] Chrikov I, Soria KM, Horgos B, Jones-White D (2020). Undergraduate and graduate students’ mental health during the COVID-19 pandemic.

[19] Clair R, Gordon M, Kroon M, Reilly C (2021). The effects of social isolation on well-being and life satisfaction during pandemic. Humanit Soc Sci Commun.

[20] Clark C, Davila A, Regis M, Kraus S (2020). Predictors of COVID-19 voluntary compliance behaviors: An international investigation. Global transitions.

[21] Cornwell EY, Waite LJ (2009). Social disconnectedness, perceived isolation, and health among older adults. J Health Soc Behav.

[22] Coroiu A, Moran C, Campbell T, Geller AC (2020). Barriers and facilitators of adherence to social distancing recommendations during COVID-19 among a large international sample of adults. PLoS One.

[23] Coyle CE, Dugan E (2012). Social isolation, loneliness and health among older adults. J Aging Health.

[24] de Bruin WB, Bennett D (2020). Relationships between initial COVID-19 risk perceptions and protective health behaviors: a national survey. Am J Prev Med.

[25] Deng Y, Chang L, Yang M, Huo M, Zhou R (2016). Gender Differences in Emotional Response: Inconsistency between Experience and Expressivity. PLoS One.

[26] Ding Y, Xu J, Huang S, Li P, Lu C, Xie S (2020). Risk perception and depression in public health crises: evidence from the COVID-19 crisis in China. Int J Environ Res Public Health.

[27] Dryhurst S, Schneider CR, Kerr J, Freeman ALJ, Recchia G, van der Bles AM, Spiegelhalter D, van der Linden S (2020). Risk perceptions of COVID-19 around the world. J Risk Res.

[28] Fong MW, Gao H, Wong JY, Xiao J, Shiu EY, Ryu S, Cowling BJ (2020). Nonpharmaceutical measures for pandemic influenza in nonhealthcare settings—social distancing measures. Emerg Infect Dis.

[29] Fornell C, Larcker DF (1981). Evaluating structural equation models with unobservable variables and measurement Error. J Mark Res.

[30] Gibson LP, Magnan RE, Kramer EB, Bryan AD (2021). Theory of planned behavior analysis of social distancing during the COVID-19 pandemic: focusing on the intention-behavior gap. Ann Behav Med.

[31] Groarke JM, Berry E, Graham-Wisener L, McKenna-Plumley PE, McGlinchey E, Armour C (2020). Loneliness in the UK during the COVID-19 pandemic: Cross-sectional results from the COVID-19 Psychological Wellbeing Study. PLoS ONE.

[32] Hämmig O (2019). Health risks associated with social isolation in general and in young, middle and old age. PLoS One.

[33] Hagger MS, Smith SR, Keech JJ, Moyers SA, Hamilton K (2020). Predicting social distancing intention and behavior during the COVID-19 pandemic: An integrated social cognition model. Ann Behav Med.

[34] Hair J, Hollingsworth CL, Randolph AB, Chong AYL (2017). An updated and expanded assessment of PLS-SEM in information systems research. Ind Manag Data Syst.

[35] Hair JF, Sarstedt M, Ringle CM, Mena JA (2012). An assessment of the use of partial least squares structural equation modeling in marketing research. J Acad Mark Sci.

[36] Hamilton K, Smith SR, Keech JJ, Moyers SA, Hagger MS (2020). Application of the health action process approach to social distancing behavior during COVID-19. Appl Psychol Health Well Being.

[37] Henseler J, Ringle CM, Sarstedt M (2015). A new criterion for assessing discriminant validity in variance-based structural equation modeling. J Acad Mark Sci.

[38] Horst BR, Sixsmith A, Simeonov D, Mihailidis A (2021). Demographic and Psychographic Factors of Social Isolation During the COVID-19 Pandemic: The Importance of Technology Confidence. Front Public Health.

[39] Jo H (2022). Effects of Psychological Discomfort on Social Networking Site (SNS) Usage Intensity During COVID-19. Front Psychol.

[40] Jo H. What drives university students to practice social distancing? Evidence from South Korea and Vietnam. Asia Pac Viewp 2022;n/a(n/a). 10.1111/apv.12351

[41] Loewenstein GF, Weber EU, Hsee CK, Welch N (2001). Risk as feelings. Psychol Bull.

[42] McInnis GJ, White JH (2001). A phenomenological exploration of loneliness in the older adult. Arch Psychiatr Nurs.

[43] Montano DE, Kasprzyk D (2015). Theory of reasoned action, theory of planned behavior, and the integrated behavioral model. Health behavior: Theory, research and practice.

[44] Nunnally JC. Psychometric theory (2nd ed). New York: McGraw-Hill; 1978.

[45] O'Sullivan R, Burns A, Leavey G, Leroi I, Burholt V, Lubben J, Holt-Lunstad J, Victor C, Lawlor B, Vilar-Compte M, Perissinotto CM, Tully MA, Sullivan MP, Rosato M, Power JM, Tiilikainen E, Prohaska TR (2021). Impact of the COVID-19 Pandemic on Loneliness and Social Isolation: A Multi-Country Study. Int J Environ Res Public Health.

[46] Palgi Y, Shrira A, Ring L, Bodner E, Avidor S, Bergman Y, Cohen-Fridel S, Keisari S, Hoffman Y (2020). The loneliness pandemic: Loneliness and other concomitants of depression, anxiety and their comorbidity during the COVID-19 outbreak. J Affect Disord.

[47] Pfattheicher S, Nockur L, Böhm R, Sassenrath C, Petersen MB (2020). The emotional path to action: Empathy promotes physical distancing and wearing of face masks during the COVID-19 pandemic. Psychol Sci.

[48] Pietrabissa G, Simpson SG (2020). Psychological consequences of social isolation during COVID-19 outbreak. Front Psychol.

[49] Podsakoff PM, Organ DW (1986). Self-reports in organizational research: Problems and prospects. J Manag.

[50] Rahman MA, Hoque N, Alif SM, Salehin M, Islam SMS, Banik B, Sharif A, Nazim NB, Sultana F, Cross W (2020). Factors associated with psychological distress, fear and coping strategies during the COVID-19 pandemic in Australia. Glob Health.

[51] Raza SA, Qazi W, Khan KA, Salam J (2021). Social isolation and acceptance of the learning management system (LMS) in the time of COVID-19 pandemic: an expansion of the UTAUT model. J Educ Comput Res.

[52] Rubin GJ, Amlôt R, Page L, Wessely S (2009). Public perceptions, anxiety, and behaviour change in relation to the swine flu outbreak: cross sectional telephone survey. BMJ.

[53] Savadori L, Lauriola M (2021). Risk perception and protective behaviors during the rise of the COVID-19 outbreak in Italy. Front Psychol.

[54] Sen K, Prybutok G, Prybutok V (2022). The use of digital technology for social wellbeing reduces social isolation in older adults: A systematic review. SSM - Population Health.

[55] Slovic P, Finucane ML, Peters E, MacGregor DG (2004). Risk as Analysis and Risk as Feelings: Some Thoughts about Affect, Reason, Risk, and Rationality. Risk Anal.

[56] Slovic PE. The perception of risk. London: Earthscan publications; 2000.

[57] Son T. HCMC to extend lockdown after September 15 to quell Covid. VNEXPRESS. 2021. Retrieved July 25th from https://e.vnexpress.net/news/news/hcmc-to-extend-lockdown-after-september-15-to-quell-covid-4355626.html.

[58] Steptoe A, Shankar A, Demakakos P, Wardle J (2013). Social isolation, loneliness, and all-cause mortality in older men and women. Proc Natl Acad Sci.

[59] Tull MT, Edmonds KA, Scamaldo KM, Richmond JR, Rose JP, Gratz KL (2020). Psychological outcomes associated with stay-at-home orders and the perceived impact of COVID-19 on daily life. Psychiatry Res.

[60] Van Orden KA, Bower E, Lutz J, Silva C, Gallegos AM, Podgorski CA, Santos EJ, Conwell Y (2021). Strategies to promote social connections among older adults during “social distancing” restrictions. Am J Geriatr Psychiatry.

[61] Victor CR, Yang K (2012). The prevalence of loneliness among adults: a case study of the United Kingdom. J Psychol.

[62] Wang C, Pan R, Wan X, Tan Y, Xu L, McIntyre RS, Ho C (2020). A longitudinal study on the mental health of general population during the COVID-19 epidemic in China. Brain Behav Immun.

[63] Wang X, Hegde S, Son C, Keller B, Smith A, Sasangohar F (2020). Investigating mental health of US college students during the COVID-19 pandemic: Cross-sectional survey study. J Med Internet Res.

[64] Wellenius GA, Vispute S, Espinosa V, Fabrikant A, Tsai TC, Hennessy J, Dai A, Williams B, Gadepalli K, Boulanger A (2021). Impacts of social distancing policies on mobility and COVID-19 case growth in the US. Nat Commun.

[65] Williams L, Rasmussen S, Kleczkowski A, Maharaj S, Cairns N (2015). Protection motivation theory and social distancing behaviour in response to a simulated infectious disease epidemic. Psychol Health Med.

[66] Yan J, Kim S, Zhang SX, Foo M-D, Alvarez-Risco A, Del-Aguila-Arcentales S, Yáñez JA (2021). Hospitality workers’ COVID-19 risk perception and depression: A contingent model based on transactional theory of stress model. Int J Hosp Manag.

[67] Zhao Y, Jiang Y, Zhang W, Zhu Y (2023). Relationship between risk perception, emotion, and coping behavior during public health emergencies: a systematic review and meta-analysis. Systems.

